# “I Don’t Know for Certain”: A Content Analysis of Reddit Posters’ Accounts of Drink Spiking

**DOI:** 10.1177/10778012251347585

**Published:** 2025-06-04

**Authors:** Jessica Ison, Erin Santamaria, Gabriel Caluzzi, Callum McAllister, Leesa Hooker, Ingrid Wilson, Jacqui Theobald, Anne-Marie Laslett, Benjamin Riordan

**Affiliations:** 1 276208 La Trobe Rural Health School, 2080 La Trobe University, Bendigo, Australia; 2 Centre for Alcohol and Policy Research, 2080 La Trobe University, Melbourne, Australia; 3 372463 Singapore Institute of Technology, Singapore, Singapore; 4 Judith Lumley Centre, Melbourne, Australia; 5 Care Economy Research Institute, 2080 La Trobe University, Melbourne, Australia

**Keywords:** sexual violence, alcohol and other drugs, social media, substance use, drink spiking, victim-survivor

## Abstract

This study analyzed drink spiking posts on Reddit, examining 91 threads and 14,284 comments to understand victim-survivor and bystander experiences. Our analysis produced four themes: contexts of drink spiking, harms experienced, immediate bystander actions, and long-term help-seeking. Drink spiking was often reported by women with male perpetrators, typically occurring in bars, private residences, or parties where alcohol was consumed and other drugs were likely administered without consent. Many victims described long-term mental health impacts and inadequate responses from emergency services. We recommend “alcohol and drug-facilitated sexual violence” as more inclusive terminology and highlight the need for interventions that validate victim-survivor experiences.

Drink spiking is understood as a person administering alcohol or other drugs to someone without their knowledge or consent, often with harmful impacts (Ison, Wilson, et al., 2024; Recalde-Esnoz et al., 2024). Drink spiking can be motivated by the intent to sexually assault, rob, harm or control a person, as the victim may be unconscious or unable to consent (Caluzzi et al., 2025). Despite frequent media accounts of drink spiking, the true prevalence of drink spiking is unclear (Colyer & Weiss, 2018). Prevalence rates are outdated and often unreliable (Taylor et al., 2004) and occurrences of drink spiking are underreported (Davies et al., 2024; Fitzgerald et al., 2017). Intense media focus may have overblown the prevalence of drink spiking (Clinnick et al., 2024; Moore, 2009; Sheard, 2011), creating a moral panic (Berrington & Jones, 2002) that may encourage people to think drink spiking is an “urban myth” (Neame, 2003). It is difficult to capture victims’ experiences due to fears of not being believed, loss of memory and shame (Weiss, 2010). There is also limited knowledge concerning why and how someone perpetrates drink spiking (Ison, Wilson, et al., 2024). This study sought to fill these gaps by understanding the nature and context of drink spiking victimization and perpetration to inform future responses. We use a novel approach of analyzing accounts on Reddit, a popular social media site.

First, a note on terminology. We are critical of the term “drink spiking,” which connotes a drug other than alcohol being given to a woman, from a stranger (Moore, 2011). We advocate for the term “alcohol and other drug-facilitated sexual violence” (AOD-facilitated sexual violence) (Ison, Wilson, et al., 2024) as it emphasizes alcohol, the most common substance used (Anderson et al., 2017, 2019), and centralizes sexual assault. However, not all incidents result in sexual violence, and drink spiking remains as the popular terminology. Therefore, we use drink spiking when discussing the popularly understood phenomenon and AOD-facilitated sexual violence when referring to academic literature. Additionally, we use terminology found when referring to particular studies, such as drug-facilitated sexual assault.

## An Overview of Drink Spiking and AOD-Facilitated Sexual Violence

Research on AOD-facilitated sexual violence is expanding. Existing studies examine toxicology, tending to focus on drug-facilitated sexual assault (Costa et al., 2020; Olszewski, 2009) and forensic legal aspects (Hall & Moore, 2008). There are limited large-scale prevalence studies focusing on drink spiking perpetration and victimization (Davies et al., 2024). Larger representative studies on sexual violence exist and commonly include some questions around alcohol and other drugs, but are not tailored to drink spiking (Basile et al., 2021; Gilmore et al., 2018; Zinzow, Resnick, Amstadter, et al., 2010). Also, many prevalence studies on drink spiking are dated. The last Australian study estimated between 3000 and 4000 incidents of drink spiking from July 2002 to June 2003, with sexual assault involved in a third of the cases (Taylor et al., 2004). A more recent national study in the United Kingdom included victims and witnesses of drink spiking (House of Commons Home Affairs Committee, 2022). It reported 1,903 cases in 2019, but focused on drink spiking only, not on associated sexual violence. Therefore, it is difficult to determine the prevalence of drink spiking and AOD-facilitated sexual violence.

Overwhelmingly, victim-survivors of AOD-facilitated sexual violence are female, and the perpetrator is male and known to the victim (Recalde-Esnoz et al., 2024). Research is limited among LGBTQIA+ communities (Hindes et al., 2025), and on perpetration. Perpetrators may act opportunistically (e.g., encourage excessive drinking until victims black out) or be proactive (e.g., predetermined administering of alcohol or other drugs without consent) (Gee et al., 2006). Known drugs used in drink spiking include flunitrazepam (Rohypnol) or other benzodiazepines, gamma-hydroxybutyrate (GHB) and ketamine (K) (Du Mont et al., 2016; Wolitzky-Taylor et al., 2008). Less understood is the role of ethanol (alcohol) in AOD-facilitated sexual violence, despite research indicating it is the most common drug used (Anderson et al., 2017; Ison, Wilson et al., 2024; Recalde-Esnoz et al., 2024). Alcohol may be added to someone's drink without them knowing, or consumption encouraged to an already intoxicated person.

Victim-survivors of AOD-facilitated sexual violence face considerable barriers to formal reporting (Cohn et al., 2013; Fitzgerald et al., 2017; Walsh et al., 2016). Reasons for this include: distrusting the police (Johnson, 2017; Orchowski et al., 2022; Zinzow et al., 2022); difficulty recalling events; internalized shame, such as believing they are to blame if alcohol or other drugs were consumed (Caluzzi et al., 2025); fear of being blamed for their own victimization (Anderson et al., 2019; Walsh et al., 2016; Wolitzky-Taylor et al., 2011); and concerns about re-traumatization when retelling their story. An additional barrier is that toxicology tests, which are often difficult to obtain, can be unreliable and provide limited legal recourse (Busardo & Jones, 2015).

Accordingly, little is known about the victim-survivor experience of AOD-facilitated sexual violence. Qualitative studies of experiences are scarce and have focused on smaller cohorts, using interviews (Brooks, 2011, 2014; Gómez et al., 2022). There are challenges in recruiting large samples, and ethical concerns around ensuring participant wellbeing and minimizing re-traumatization. Moreover, media reports of individual cases that reach public discourse run the risk of sensationalizing events and experiences (Clinnick et al., 2024). Thus, our understanding of AOD-facilitated sexual violence is limited. Our study seeks to fill this gap in the literature by providing a detailed overview of victim-survivors’ perspectives.

## Social Media as a Source of Victim Accounts

Social media platforms like Reddit provide useful data for researching phenomena like drink spiking. Social media sites have become extremely popular; people spend an estimated 151 min per day on social media (Dixon, 2024) and there are over four billion global posters (people who post). Reddit is particularly popular, and consists of “subreddits” or topic-specific forums where people can post stories and interact with others’ stories (by commenting on or upvoting posts). Though widely used, Reddit does have an oversaturation of white American males who post on the site (Pew Research, 2016; Statista, 2023). A defining feature of Reddit is that posters often use pseudonyms and remain anonymous. This anonymity enables Reddit posters to discuss certain topics that may be stigmatizing with less fear of judgement (e.g., struggles with substance use; Mason et al., 2021). Researchers have increasingly used Reddit to explore people's experiences and perceptions of stigmatized social practices and problems, including to forward suicide theories (Mason et al., 2021) and identify the support people receive when recovering from addiction (Garg et al., 2021; Valdez & Patterson, 2022).

Sexual violence victim-survivors also use social media to disclose experiences and seek support (Fileborn & Loney-Howes, 2020; Schwab-Reese et al., 2025)–likely enabled by the barriers they face to formal disclosure and help-seeking (Corbett et al., 2023; Loney-Howes, 2020; Zinzow et al., 2022). These experiences shared on social media can produce detailed narratives that are difficult to obtain through conventional qualitative research. In particular, since the advocacy of Tarana Burke and the #MeToo movement, social media has provided an avenue for open disclosures of sexual violence (Alaggia & Wang, 2020; Fileborn & Loney-Howes, 2019), although the voices of minority groups remain, at times, excluded (Ison, 2019; Phipps, 2019). Research has examined attitudes of sexual violence victim-survivor posts on Reddit, finding that victim-survivors can feel sexual assault stigma (Lanthier et al., 2023) and have negative experiences of formal help-seeking (Abavi et al., 2020). Thus, Reddit has provided a supportive community where they can freely share their stories and seek advice (O’Neill, 2018). However, to date, no Reddit research has focused on drink spiking accounts.

## Overview of the Current Study

There are clear gaps in the research on drink spiking and AOD-facilitated sexual violence. These include details around the contexts and substances people perceive as pertinent, and methodological lines of enquiry that draw on large-scale samples. This study aimed to address these gaps and inform social policy responses by examining experiences of drink spiking through analysis of victim and bystander accounts on Reddit.

This project is framed by a critical feminist approach to understanding sexual violence as culturally embedded. This approach understands that sexual violence is a highly gendered phenomenon that particularly impacts women and LGBTQ+ communities (Ison et al., 2025). Understanding this cultural phenomenon is essential for developing a primary prevention approach (Hooker et al., 2020) that addresses all aspects of the issue across the socio-ecological continuum (Heise, 1998).

## Methods

This descriptive qualitative study uses content analysis of Reddit posts. The steps are outlined below.

### Data Collection

We began by defining search terms and scraping relevant data from Reddit. To identify relevant threads and comments, the research team agreed on the search terms “roofie*,” “drug*,” “date rape*,” “drink spik*” with the term “Reddit” and Ison and Riordan searched Google (similar to Mason et al., 2021). Reddit's search function and the search function from the RedditExtractor package in R yielded a lot of irrelevant threads (see below for a description of RedditExtractor). Therefore, we opted to use Google search to identify threads on Reddit. Each thread identified in the Google search was opened and screened against our inclusion criteria, which stated that: (1) the thread was in English, (2) the thread included at least one comment, and (3) the post was broadly about drink spiking (where drink spiking was used as expansively as possible, including our understanding of AOD-facilitated sexual violence as well as spiking that did not include sexual assault). Any threads that did not match the inclusion criteria were excluded. For each search, we screened all the Google results until the Reddit posts were exhausted (i.e., we stopped when the search no longer recommended Reddit threads). The URL was recorded for any relevant thread, and a sample of 91 independent threads was included after 10 duplicates were removed.

**Figure 1.**
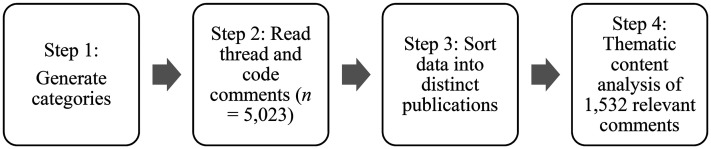
Data analysis.

Once all the threads were identified, we used R and the r package RedditExtractor (Rivera, 2023) to interact with Reddit's Application Programming Interface (API) to collect all posts on each thread for analysis. RedditExtractor allowed us to collect information about the thread (e.g., title, text, number of comments) and the comments on the thread (e.g., text, upvotes, order of post in the thread). At the time of extraction, Reddit's API allowed us to collect 500 posts per thread (meaning only the first 500 posts on a thread can be collected). Once data were collected and merged, we used R to generate a globally unique identifier (GUID) for any poster's name and removed the poster names from the dataset to de-identify the poster. Finally, the data were exported as an Excel file for coding and analysis and consisted of 14,284 comments from 91 threads (Figure 1).

### Data Analysis

Data analysis involved four steps, with a two-part content analysis approach (steps 2 and 4) outlined by Prior (2014). Content analysis has also been used elsewhere for social media analysis (e.g., Mason et al., 2021). Here, it was undertaken by three research team members (Ison, Santamaria, and McAllister). We chose a qualitative methodology because we did not seek an objective “truth” in the data. Rather, we aimed to generate themes that represented reoccurring patterns (Braun & Clarke, 2022).

Step one involved categories aspects of the drink spiking incident, including: (a) victim information (such as gender); (b) perpetrator information; (c) nature of the assault (such as substance, sexual assault and other crimes); (d) setting; and (e) impacts and effects of the incident. It also involved categorizing responses to drink spiking, including: (a) negative responses; (b) supportive responses; (c) suggestions for the victim (such as people they could talk to or reporting to police).

Step two involved first reading the original post (the thread). The original posts were diverse, including personal accounts, questions (e.g., “anyone had a spiked drink? What happened?”), media articles about drink spiking or a thirdhand account of an incident (e.g., “OMG!!! My friend was spiked!!!”). The original threads content only had to relate to drink spiking and have generated conversation. After reading the original thread, we next deductively coded the comments. The three authors who coded the data coded simultaneously so conversation and clarification could happen throughout the process, with the lead author having the final say. Coding together also ensured that researchers could debrief, as content was distressing, and researcher well-being was important to monitor with self-care strategies employed to support the team (Sexual Violence Research Initiative, 2015). Once deductive coding was complete, the lead author cross-checked 10% of the codes. Each post was read at least once to assign any relevant categories. Once completed, 5,023 of the posts had relevant content and were assigned categories. Each post could have multiple categories it was coded to, and a single comment could include multiple incidents.

At times inference was needed during coding. For example, gender could be inferred if someone referred to their clothing (e.g., “My bra was missing” or “I remember touching a penis”). While this has the potential to misidentify posters who were gender-nonconforming, there was often enough evidence to indicate the posters’ self-identified gender. Another example is inferring incident location. For example, “we were out drinking on the weekend” was assessed as meaning in a bar or club.

Step three involved deciding on how data would be organized for publications. Given the large data set, the authors agreed that the data would need to be separated into multiple articles for depth of analysis and discussed possible topics for analysis. For this article, we agreed to focus on disclosures of drink spiking, including first-hand accounts (i.e., direct victims) and bystander accounts (i.e., witnesses). Some spoke about being spiked with another person, so they were coded as both a first-hand and bystander account (i.e., “me and my friend were spiked”). When people made disclosures on Reddit, others sometimes comment on their disclosure. These revealed various attitudes that will be reported in a separate analysis. Therefore, for this article, we had 1,532 posts that were first-hand accounts or bystander accounts (or both).

Step four involved thematic content analysis to develop themes. Authors one and two generated the themes, then author one sorted the data into those themes. The themes we used for this paper are, “The contexts of drink spiking,” “The harms of drink spiking,” “Immediate bystander responses,” and “Long-term help-seeking and response.”

### Ethical Considerations

Internet research offers unique possibilities to analyze large data sets, but comes with ethical concerns (franzke et al., 2020). While Reddit posts are typically anonymous, researchers can further ensure anonymity, particularly as consent to use their data cannot be obtained. We engaged multiple strategies to follow ethical guidelines employed in social media research (Colditz et al., 2018; O’Callaghan & Douglas, 2021). We discussed whether extracting data from Reddit was an acceptable and ethical form of research, particularly given the likelihood of extensive personal accounts of drink spiking. We had been researching drink spiking for some time, and struggled to recruit participants (in part due to resourcing constraints). We were earnest in our hopes to learn drink spiking trends from a large data set. We looked to social media research to assess whether such research is ethical for analyzing drink spiking and found no consensus (O’Callaghan & Douglas, 2021). We decided that the benefits outweighed negatives given the lack of existing research, difficulties and ethical risks in collecting first-hand data, and the clear implications for victim-survivors and prevention programs. However, we committed to following clear ethical guidelines drawn from other researchers and our own discussions.

First, the first and last authors designed the study. Ison has experience in sexual violence research and, in particular, qualitative research including interviewing victim-survivors (e.g., Ison, Forsdike, et al., 2025) and feminist critical discourse analysis (e..g., Clinnick et al., 2024). She has also undertaken training in sexual violence disclosures. Riordan has researched social media and published findings with careful ethical consideration (e.g., Mason et al., 2021). The design was presented to the broader team who have extensive research knowledge and experience, including in sexual violence prevention, qualitative methods and community engagement.

Second, we applied for ethics and used the process to rigorously consider the ethical imperatives of such research. The study was approved by the La Trobe University Human Ethics Committee (HEC 23045). Third, we decided to distil a broad overview of the data rather than undertaking any in-depth analysis of single posts. Our aim was not to provide quantitative analysis but to understand the patterns and themes in the data. As such, we utilized principles of ethical fabrication (Markham, 2012). This included changing key words (e.g., changing “I had a vodka” to “I had a gin”) as well as amalgamating multiple similar posts (e.g., amalgamating “My drink was spiked on the weekend” and “I had a spiked drink on Friday night” to “On the weekend someone spiked my drink”). Such an approach adheres to the amalgamated meaning of many posts while minimizing possibility of identifying individual posters through back searching on the internet.

However, we have one caveat where we opted for a more generalized description. Sexual assault was described in different levels of detail by posters, and some used graphic descriptions. We have refrained from providing these. We made this decision with caution, as we did not wish to silence any victim-survivors, but were equally concerned that graphic descriptions could be difficult for readers and risk being voyeuristic.

Fourth, we decided not to make our data open access. While some research benefits from having data sets publicly available, we decided this would be unethical with this data set because it could allow the posters to be identified. Fifth, we wanted to make sure our research goes beyond academic publication to be accessible and useable for the community and in particular victim-survivors. As such, we have a dissemination plan that includes non-academic outputs and presenting to the sexual violence sector.

### Reflexivity Statement

The research team is interdisciplinary and have worked together on multiple sexual violence projects in different capacities. However, we come with our own biases and assumptions. Most important to note is the three of us who coded the data. All three of us are white, two are female, and one is male. We have different educational backgrounds including Ison holding a PhD, Santamaria holding an Honours and working on this project as a research assistant and McAllister working on this project for his Honours project. As such, while we attempted to minimize bias, there were still power imbalances between those coding. Also, it is important to note the emotional toll reading this data had on the three of us, including vicarious trauma. We employed self-care strategies, such as taking regular breaks and talking with a counsellor to mitigate vicarious trauma, but it still likely impacted how we coded the data. Having these experiences resulted in extensive conversation on how we might shape the research differently in future to reduce vicarious trauma.

## Results

This section presents an overview of the data before discussing the four key themes (Table 1). Posters tended to either describe their own experience, or situations where they were a bystander (i.e., their friend was spiked and they got them home to safety). Common patterns were found across posts, notably regarding the victim's and perpetrator's genders. As noted, some inference was needed when assessing the gender of the victim or perpetrator, and it was not always possible. However, where noted or inferred, overwhelmingly, the victim tended to be female and the perpetrator male. In contrast, a common narrative in the data was that men were not the intended target of the drink spiking, but inadvertently consumed spiked drinks meant for a female partner or friend: “I was spiked once, and I’m a guy. My friend (female) didn’t want her drink, so I had it and then almost passed out. Just glad it didn’t happen to her.”

**Table 1.** Themes.

The contexts of drink spiking
The harms of drink spiking
Immediate bystander responses
Long-term help-seeking and response

There were limited posts where people identified themselves as LGBTQ+. We did not count scenarios where the perpetrator was the same gender as the victim unless they were explicitly identified as LGBTQ+. For example, “I was chatting with this guy (I’m bi) and left my drink unattended. Later, felt out of it.” It was noted by some of these posters that drink spiking is not only something heterosexual women experience, “It happens in queer events too.” Such an occurrence could be difficult if victims were not open about their sexuality: “I finally came to terms with being gay… went to a bar to make friends… Chatted to a man… I was sexually assaulted… I didn’t report because I was closeted.”

Alongside the similarities in the reported gender, there were some similarities in the relationships between the victim and the perpetrator. Many accounts suggested an unknown person was the perpetrator. For example, many posters described getting an alcoholic drink from a bar and at some point, feeling sick or losing consciousness. They would then speculate who the perpetrator was such as “it must have been the bartender” or “A man was talking to me and I turned away for a second.”

While it was often difficult to assess who the perpetrator was, there were also many accounts where the poster identified the perpetrator. The perpetrator could have been known to the victim intimately, such as a partner: “It happened to me from my boyfriend and his friends,” or someone they had just met: “I met this guy for a first date. It was going well. I had a few drinks. Next thing I wake up in my room sobbing.” Another common suggestion was that the perpetrator was the bartender: “Me and a friend had it happen months apart, at the same bar with the same guy.” Friends were also discussed by many posters: “My ‘friend’ roofied me at a party” or “I was drugged and assaulted by one of my best friends.” The last reported known perpetrator was a colleague or boss: “I was drugged by my boss at a party.”

When the perpetrator was a stranger, the poster would identify the perpetrator by explaining how they knew the person had committed the assault: “It was the bartender, I saw the footage” or “My friend was dropped at a train station [after the assault] and the idiot was caught on camera. He is in jail now.”

### The Contexts of Drink Spiking

Posters identified contexts that surrounded drink spiking incidents (Table 2). These included immediate conditions such as the location of the incident, or substances involved, both voluntary and involuntary.

**Table 2.** Contexts sub-themes.

**The contexts of drink spiking**
Location
The substances
AOD voluntary consumption

#### Location

Although sexual assault or other events proceeding drink spiking happened in different locations, most posters reported that the drink spiking incident occurred at a licensed venue (where alcohol could be bought and consumed on site). These venues included hotel bars, pubs, clubs where people could watch erotic performances, nightclubs for young people and small local pubs. There was no one specific type of licensed venue that was discussed.

Outside of bars, pubs, and clubs, some private venues were consistently discussed. One common location was a house party, which included intimate parties: “At a small party, I blacked out after three drinks” or larger parties with many unknown people: “We had never met the person throwing the party before.”

Within the party setting, posters talked about seemingly targeted attacks: “He insisted we have shots [of alcohol].” This could also include multiple people who were engaged in the incident: “There was six guys at the party… I woke up with no clothes on.” These examples show that perpetration did not only occur in licensed venues. Though, there were also examples where it seemed the perpetrator acted opportunistically: “Then I passed out in one of the rooms upstairs… Remember a man on top of me.”

Some of the posters also described university or college parties or events. These were both events organized within the university context and events where they were at a local university bar or hang out. Included in these descriptions were parties held at sororities or fraternities, which are a type of college social group common in the U.S.: “I was spiked at a frat party.”

While the spiking itself may have occurred at a public venue or private house party, if victims were sexually assaulted it often occurred at a person's home. Posters rarely reported having a memory of how they were taken back to the house. There were also examples where posters were at someone's house, after a date or spending time with someone they considered a friend. At some point, the person or people administered extra alcohol or other drugs to them: “I was in his apartment alone… He slipped me GHB” or “At a ‘friends’ place… They spiked my drink… I woke up being raped” or “I was studying with another student at my place… He gave me a juice… I remember him assaulting me.”

Less commonly reported locations for drink spiking included public places like alleyways or sporting events and music festivals or raves: “I was at a festival… I don’t know if I was spiked but I was suddenly out of it.” Another lesser identified location was work-related contexts, such as work parties.

#### The Substances

The most identified drugs were GHB and ketamine. Without a toxicology test, it was difficult for posters to know what substance they had consumed. Those with a toxicology test generally identified GHB or ketamine. Some posters simply stated they were “roofied,” a colloquial term for Rohypnol. Though there were examples of Rohypnol in toxicology reports, “roofie” was a catch-all term where drink spiking was suspected. Some posters also mentioned alcohol being administered to them without their knowledge: “I found out he put alcohol in my hot chocolate,” someone giving them extra alcohol without their knowledge: “I saw a guy put a shot into my friends drink” and encouraging them to continue drinking: “He kept pouring me huge glasses of wine and pressuring me to drink them.” Less commonly, other drugs mentioned included party drugs colloquially called MDMA or ecstasy (i.e., 3,4-methylenedioxy ​methamphetamine), hallucinogenic drugs colloquially called LSD, pharmaceutical drugs such as valium and opiates, marijuana, phencyclidine (PCP), and methamphetamine.

After discussing the effects the drugs had on them, posters sometimes asked others to help identify what drugs they were. For example, some talked about feeling as though they were “in a fantasy” and “outside my body,” which other posters would identify as GHB. While not definitive, such discussions gave posters some sense of understanding: “After reading this thread… I think it was definitely GHB.”

#### Alcohol and Other Drug Voluntary Consumption

In general, posters talked about consuming alcohol when a drink spiking incident occurred. For example, “I got a drink from the bar and after a few sips I felt dizzy” or “Yeah had a drink spiked once.” Some discussed their alcohol consumption to prove their drink had been spiked: “I had only one drink and I passed out” or “Sure, I’d drunk heaps, but at the time I was basically an alcoholic, I would never pass out from that much alcohol” or “I had two shots before I went to the party. I also had dinner. When I got to the party I had one beer and felt so sick.” To a lesser extent, posters would mention other drugs they willingly consumed prior to the drink spiking event.

### The Harms of Drink Spiking

#### Sexual assault

Overwhelmingly, the descriptions of the sexual assaults reported after drink spiking indicated a cisgender male sexually assaulting a cisgender woman through vaginal penetration. Other forms of sexual assault were discussed by posters of any gender. This included anal penetration, which included descriptions of anal rape or allusions to this: “I felt lube in my butt cheeks.” While most identifiable sexual assault victims were female, anal penetration was also disclosed by men from a male perpetrator (Table 3).

**Table 3.** Harms sub-themes.

**The harms of drink spiking**
Sexual assault
Robbery
Physical and psychological effects
Memory loss (or blackouts)

Sexual violence was described in different levels of detail. While some posters provided graphic descriptions, other posters implied a sexual assault: “The next thing I remember… both of us naked, you can figure out the rest.” Posters also provided a general summary of what happened: “I was drugged and raped by my ex-boyfriend.” Or when describing what happened posters often offered generalized details: “Someone had sex with me when I was passed out” or “The next thing I knew, I woke up and he was sexually assaulting me.” They also described the perpetrator's actions in a manner that alluded to their sexual assault: “All of this [impact on me physically and mentally] so this arsehole could have a fucking orgasm.”

They might have limited memory beyond: “I remember saying no… woke up naked.” Posters also extended this to voicing that they thought they were sexually assaulted: “I blacked out, I think I was raped,” though they may only have a suspicion: “I don’t know for certain.”

Others reported not realizing it was sexual assault until many years later: “I was at a friend's place once and he gave me very strong drinks until I was drunk, then pressured me to have sex. Years later someone told me this was rape.”

Posters described being orally penetrated, including by one or multiple men. Some posters also described a gang rape where multiple perpetrators assaulted them: “I was 14 and hanging out with some older men. It made me feel tough. I remember taking a drink from them. And then I remember only some snippets. That's how I lost my virginity.” Some of these cases also involved video recording: “I remember there was three guys. They had a camera.”

While uncommon, some posters described being forced to penetrate someone else, which could also be paired with a description of people watching the sexual assault.

Posters also described scenarios where someone attempted to sexually assault them and they escaped: “Someone spiked my drink, I somehow ran away” or a friend intervened: “My boyfriend found me passed out with some guys trying to take me out of the venue, he got me away from them and took me home.”

Many posters managed to avoid sexual violence and make it home safely. However, they still experienced ongoing distress and fear from the spiking, indicating that spiking alone was a traumatic experience, as well as the sexual assault (when it happened), as discussed further below.

When some posters joined a conversation on a thread to talk about a specific experience or experiences of drink spiking, they commonly disclosed other forms of sexual violence they had experienced. For example, one participant explained why she did not report, saying “I already had PTSD [post traumatic stress disorder] from my ex who was abusive… I didn’t believe my memories.”

#### Robbery

Posters also described situations where their belongings were stolen from them. Robbery included times where the perpetrator's intention was clear and proactive: “I came to on the street without my wallet or phone” or “I was beaten and robbed.”

There were also situations where the robbery or attempted robbery was opportunistic: “I was at a bar… I started to lose consciousness… A girl [I tried to get help from] she tried to rob me.” Robbery might also be something that happened alongside sexual assault. One person describing having his drink spiked at a bar and getting in a taxi with a man and “He robbed my apartment and sexually assaulted my roommate.”

#### Physical and psychological effects

Posters talked about not being able to move during the period they were substance affected: “I legit couldn’t move” or “My motor skills stopped.” Posters also described feeling as if they were “in a fog” or “not in my body.” They also discussed vomiting, likely from the substance or the combination of substances: “she said I was vomiting profusely” or “I vomited everywhere.” Posters might have also had physical impacts when they regained consciousness such as “grazed knees” or “a stiff neck” or “I peed my pants” with little memory of how it happened.

Immediately post incident, posters talked about feeling sick or having a headache: “I woke up with a slamming headache” or “I woke up with the worst hangover.” This feeling could last for days “I felt like I had the flu for the next few days.” Though, others used their lack of hangover as proof they were given drugs when they had been drinking: “I vomited and then I was totally fine, like sober.”

Posters described having to go to hospital due to the negative interaction between prescription medicine medication (such as epilepsy medication) and illicit substances: “I had seizures.” In extreme cases, posters had lifelong physical consequences: “I have to walk with an aide now [due to the sexual and physical assault].” Overwhelmingly, posters frequently had subsequent anxiety and poor mental health, such as being afraid to go out: “I never went clubbing after that” or “NEVER went out again”; not trusting people: “you can’t trust anyone with your drink”; and mental health struggles: “It fucked with her head” or “I struggled with panic attacks for a few years” or “I have never been so low. I still can’t make friends.”

People experienced ongoing psychological effects from drink spiking regardless of whether it resulted in sexual assault. While it is clear a drink spiking incident alone is traumatic, posters would voice gratitude that it “wasn’t worse” and yet often still list many of the ongoing psychological effects they experienced.

#### Memory loss (or blackouts)

Regardless of whether a poster was sexually assaulted or not, memory loss (or blackouts) was frequently reported when people posted about their experience of drink spiking. The length of memory loss varied, with some reporting having flashes of memory: “I’ve got brief flashes of being loaded into a taxi” while others reported complete memory loss for longer periods of time: “I woke up six hours later” or “I have no memory of what happened after [having one drink].” Memory loss was also paired with confusion over what happened. As mentioned above, this may have included a suspicion they were sexually assaulted. For those who were not sexually assaulted, some did not remember what happened and relied on friends filling in their memory: “In the morning… they laughed and told me what I had done the night before” or “they told me nothing I was saying was making sense [the night before].” Posters would try to piece their night together from different people's accounts. However, they often felt the ongoing effects of this memory loss: “It still haunts me that I don’t know what happened” or “9 years later and still no memory of what happened… Fuck, I feel sick at the thought.”

### Immediate Bystander Responses

Bystander responses were discussed in two ways. First, posters would talk about bystanders intervening before they were spiked. The most common occurrence was stopping them from consuming their drink that had a substance in it: “When I worked at a bar I caught a bunch of men trying to spike some girls drink. I always went straight over and told her.”

Second, posters described protective factors undertaken by bystanders designed to stop a sexual assault from happening after someone was spiked. For example, where a friend recognized what had happened they might take them home: “thankfully my friend got me home.” They similarly talked about a partner, family member or acquaintance who helped them. Posters also had assistance from strangers, which stopped a potential sexual assault. These included bar staff or security: “bar staff called the cops,” though bystanders could also be strangers who found them in a toilet or on the street and helped them to safety: “the girl in the next cubicle called an ambulance,” “a girl took my arm and led me outside… woke up the next day safe at her place… could have been so much worse.” In their role as bystanders, people appeared to both identify signs of drinking spiking, as well as provide support to victims: “I could tell something was wrong with my wife… took her home.”

### Long-Term Help-Seeking and Response

Posters identified formal and informal responses that could be both positive and negative. They talked about going to the police but, in general, described little action being taken: “Reported to the cops, nothing happened,” “made a statement and everything, didn’t hear from them again” or active dismissal from the police: “Tried to report being raped and spiked, the police didn’t care.” In some accounts police also blamed the victim: “We took her to the cops. They told her she must have liked being [physically abused] that way.” There were very few positive encounters with the police shared by posters.

In addition to experiencing negative interactions with the police, another barrier to reporting was victims not knowing what happened to them: “I tried to explain it to the police and doctors, but I didn’t really know what happened.” Such memory loss also extended to seeking help from medical professionals, particularly in emergency departments. Posters described being too intoxicated to explain what happened and, as a result they were dismissed: “Even though my friends tried to tell them I was never usually like this, they refused to do a test, said I was just drunk.” By contrast, posters recounted many examples of health care staff as hospitals taking them seriously: “Woke up in the hospital. They tested me and found GHB.”

There were also discussions concerning the response from the venue staff, if the incident occurred in a public licensed venue. Some venues were described as helpful at the time of the assault, such as by getting the person safely back to their friends, as well as afterwards, by providing footage: “They gave the police footage and you could see the guy doing it.” Others reported staff to be unhelpful and denying assistance: “The bar threw me out, so I wasn’t sick on their premises.”

One reason posters identified for not formally reporting or informally disclosing was due to blaming themselves for their drink being spiked: “I asked a drink from him… I know, I know” or “I shouldn’t have had the drink.” They would also take on self-blaming attitudes and offered justifications for what they viewed as their role in the incident: “I know I was spiked because I wouldn’t sleep with him sober, I was a virgin.” They would also note the blame they felt for periods after the assault: “I thought it was my fault for years.”

## Discussion

To date, limited qualitative research has examined situational experiences and understandings of drink spiking. Our analysis of Reddit posts partially fills this gap by looking at accounts of drink spiking, which are likely shaped by broader cultural narratives. As such, it aligns with some of the existing research on drink spiking and AOD-facilitated sexual violence, where most available accounts are from women (Recalde-Esnoz et al., 2024). In the data, when men disclosed being victims of drink spiking, they often reported having accidentally taken the drink of the intended victim. Those who reported being sexually assaulted often identified a male perpetrator. There were limited people who identified themselves as trans or gender-diverse. This could indicate that trans and gender-diverse people are not comfortable posting on Reddit, likely because they anticipate greater negative social reactions (Edwards et al., 2023).

While sexual assault was central to most accounts of drink spiking, there were a range of other harms described by victims. These included theft, physical and mental health effects, a sense of self-blame, and memory loss (both blackouts and fragmentary memory loss). Research on sexual violence shows that victim-survivors can have worse PTSD impacts if they experience memory loss (Fields et al., 2022; Zinzow, Resnick, McCauley, et al., 2010), indicating a need to further examine drink spiking and memory loss. Such research should also consider the harm caused by the spiking itself, as well as sexual assault when it occurs. It is important that health and social responses to drink spiking acknowledge and account for these harms to victims.

Our findings support the research on sexual violence facilitated by alcohol and other drugs, which shows that perpetrators are most likely male and often known to the victim (Anderson et al., 2017; Badour et al., 2020; Ison, Wilson et al., 2024; Prego-Meleiro et al., 2021). However, strangers were regularly reported or suspected as the perpetrator, which aligns with common drink spiking narratives (Clinnick et al., 2024). While we cannot speak to the motives of perpetrators, the administration of alcohol and other drugs to victims seemed to occur both proactively and opportunistically (Gee et al., 2006). Proactive perpetration was characterized by the use of depressant, sedative or dissociative drugs such as GHB or ketamine, which required a degree of preplanning. Opportunistic perpetration was characterized by alcohol use in environments where alcohol was readily available. Alcohol was often involved in drink spiking events, where the victim and at times the perpetrator were drinking, often in a bar or a club. The central role of alcohol in facilitating sexual violence, both in terms of alcohol-centered contexts and its use to facilitate intoxication of the victim, points to the need for further analysis of alcohol's role in facilitating sexual violence (Abbey, 2011).

Bars or clubs were common settings discussed, despite research indicating that the most likely setting for drink spiking is a private residence (Anderson et al., 2019; Swan et al., 2017). One possibility for this disparate finding is the use of the terminology “drink spiking” in our search terms. The general understanding of drink spiking is a stranger administering drugs in a bar or club, so it follows that posters would disclose experiences that fit this narrative when discussing drink spiking. While posters would discuss a bar or club as the setting where they suspected the drink spiking occurred, sexual assaults were also described as being perpetrated in a private residence or a place away from the bar or club (alleyway, etc.). There were also considerable reports of the location being a private residence, such as after a date, or a house party. Greater awareness of how different locations might facilitate different aspects of drink spiking and sexual violence could improve reporting and shift the common narrative of strangers in bars or clubs.

Improved awareness about drink spiking could also be extended to emergency services. Posters often relayed negative stories of interactions with police, which is reflected in the research on drink spiking (Clinnick et al., 2024; Cohn et al., 2013) and sexual violence broadly (Stewart et al., 2023). Considering these negative interactions with emergency services, posters talked about the positive support they received from friends when they disclosed. Evidence shows that informal supports are crucial for sexual violence victim-survivors broadly (Zinzow et al., 2022), which is reflected in the poster's accounts.

Posters also talked about the important role of bystanders as a protective factor, particularly preventing spiking from occurring or responding afterwards. The important role of bystanders has been discussed in relation to sexual violence (Mainwaring et al., 2023). Bystander interventions have faced scrutiny as to their effectiveness (Porat et al., 2024) for overlooking how intoxication impacts bystander behavior (Leone et al., 2018). Given the central role of bystanders in this study, it is possible that they play a unique role in relation to drink spiking that warrants further investigation.

### Strengths and Limitations

This study was conducted with data from anonymous posts on Reddit. Therefore, it is not possible to assess the veracity of the posts. The findings may also not be generalizable as it only accounts for those who wish to share their story on Reddit. Nonetheless, research using social media is an increasingly common method as it allows for large quantities of data to be analyzed, and this anonymity is also a strength as posters may be more forthcoming than they otherwise would be. Also, while interviews are an important form of data collection, they have the potential to cause harm, which this form of data collection avoids. Another issue is that it is difficult knowing the geographical origins of the posts. While some posters may identify a specific location such as a country, many did not. Therefore, we have limited ability to draw conclusions on drink spiking in any specific locations. It is likely that posters were predominantly from the US given the large number of North Americans on the site (Shatz, 2017; Statista, 2023). Knowing the location might allow for more specific knowledge on the local contexts and laws, which could help with advocacy and prevention. Nonetheless, an overview of drink spiking is an important step toward deepening our understanding this harmful social practice and how drink spiking narratives operate. Future research could look at country-specific data to advocate for tailored prevention initiatives.

Further limitations of Reddit include that it only captures people who are comfortable posting on this platform, potentially excluding people in countries who do not have access to Reddit. Older groups, women, people with less education, and people of color are less likely to use Reddit than other groups (Pew Research, 2016). Also, posts were in English, so important data in other languages may have been missed. Future research should consider how to engage more diversity in terms of location and language spoken. However, we were still able to gather over 14,000 posts, which is one of the largest data sets available on drink spiking. Last, while we are critical of the term drink spiking, we have nonetheless used it throughout this paper as it reflects popular understandings of alcohol or other drugs used to facilitate sexual violence. While we advocate for broadening this term to AOD-facilitated sexual violence to be more expansive, we inadvertently also lend credibility to the term drink spiking in using it throughout the paper. However, one of the strengths of this paper is drawing attention, through a critical feminist analysis, to the issue of drink spiking, which also opens an opportunity for advocating for a more expansive terminology, such as AOD-facilitated sexual violence.

### Implications for Future Research

This study contributes to the knowledge on drink spiking and the broader concept of AOD-facilitated sexual violence. It shows that, despite concerns that drink spiking is sensationalized in the media, the impacts on victims and the continued victim blaming and stigma have very real implications. Future research should consider intervention, prevention, response, and policy. Intervention could include challenging stigmatizing narratives of drink spiking. Further, interventions could consider how to harness popular forums such as Reddit as a tool for education and change. Interventions to address drink spiking should go beyond looking at the behavior of individual women to considering the social context, including how this occurs in many locations, with many different types of perpetrators.

### Prevention and Policy Implications

Prevention should include promoting a broader understanding of AOD-facilitated sexual violence through community education and social marketing campaigns. Prevention interventions should thus have a focus on preventing perpetration, while considering the central role of facilitators like alcohol (Hooker et al., 2021). In terms of response, future research should also examine emergency service response to understand the experiences of victim-survivors seeking help. Policy on drink spiking is to date under explored and future research could map global drink spiking policy and identify key barriers to change.

## Conclusion

Our research draws on disclosures of drink spiking on the platform Reddit to highlight the various contexts, harms, and responses to drink spiking. The posts show that drink spiking has serious implications for victim-survivors, both short- and long term, and that future research needs to address these impacts and develop approaches to prevention and response.
